# Mixtures, metabolites, ionic liquids: a new measure to evaluate similarity between complex chemical systems

**DOI:** 10.1186/s13321-016-0159-x

**Published:** 2016-09-22

**Authors:** Andrea Mauri, Davide Ballabio, Roberto Todeschini, Viviana Consonni

**Affiliations:** Milano Chemometrics and QSAR Research Group, Department of Earth and Environmental Sciences, University of Milano-Bicocca, P.zza della Scienza, 1, 20126 Milan, Italy

**Keywords:** Hausdorff distance, Chemical mixtures, Metabolites, Hausdorff-like similarity, QSAR

## Abstract

This communication deals with the scientific problem of evaluating the similarity between two chemical systems, each described by a finite discrete set of elements/members, which are in turn *p*-dimensional vectors of chemical/biological descriptors. A variant of the Hausdorff measure, called Hausdorff-like similarity (*Hs*), is proposed aimed at taking into account information on all the elements present in the compared sets, information that is usually lost by the other measures.

In Quantitative Structure–Activity Relationships (QSARs) studies, several modelling strategies are based on the calculation of similarity/diversity measures between molecules with the aim to predict the unknown properties of a target from the known properties of the most similar molecules [1]. The evaluation of toxicological and environmental impact of chemical mixtures, ionic liquids and molecule metabolites through computational approaches like QSARs shifts the focus from the analogy relationships between single objects, which commonly are molecules, to those between sets of objects, which can be the substances of chemical mixtures, the metabolites or molecular substructures that are generated by molecule dissection, or the different ions that form ionic liquids.

This communication deals with the scientific problem of evaluating the similarity between two chemical systems, each described by a finite discrete set of *p*-dimensional elements, *p* being the number of variables that are used to describe each element of the sets to be compared. Despite this communication is focused on chemical problems, the same theory and considerations apply if one compares social networks characterized by groups of users or research groups composed of researchers who are described by different bibliometric indices.

Among the huge number of similarity/diversity measures [2] proposed in the literature, functions suitable for evaluating the proximity between sets are the Hausdorff distance and the linkage metrics used in hierarchical clustering methods. In this note, a variant of the Hausdorff measure [3], called Hausdorff-like similarity (*Hs*), is proposed aimed at taking into account information on all the elements present in the compared sets, information that is usually lost by the other measures.

Let A and B be two nonempty finite sets of $n_{A}$ and $n_{B}$ elements (e.g., chemical structures or sub-structures, molecule metabolites, ions, mixture compounds, etc.), respectively, each element being described by *p* variables (e.g., chemical or biological descriptors). Then, let *s* be a similarity measure between two elements in the *p*-dimensional space, which is a measure of the proximity strength of the two elements and takes values in the range from 0 (minimum similarity) to 1 (maximum similarity).

The Hausdorff formula [3] for measuring the diversity relationship between the two sets A and B is defined as:

$$dHaus_{AB} = \hbox{max} \left\{ {\mathop {\sup }\limits_{a \in A} \left[ {\mathop {\inf }\limits_{b \in B} \left( {d_{ab} } \right)} \right],\mathop {\sup }\limits_{b \in B} \left[ {\mathop {\inf }\limits_{a \in A} \left( {d_{ba} } \right)} \right]} \right\}$$

from which, the corresponding similarity measure can be calculated as:

$$sHaus_{AB} = \hbox{min} \left\{ {\mathop {\inf }\limits_{a \in A} \left[ {\mathop {\sup }\limits_{b \in B} \left( {s_{ab} } \right)} \right],\mathop {\inf }\limits_{b \in B} \left[ {\mathop {\sup }\limits_{a \in A} \left( {s_{ba} } \right)} \right]} \right\}$$

where the symbols *d* and *s* refer to the distance and the similarity measures, respectively.

The novel Hausdorff-like similarity *Hs*_*AB*_ between the two sets A and B is defined as:

$$Hs_{AB} = \frac{{\sum\limits_{a \in A}^{{}} {\mathop {\hbox{max} }\limits_{b \in B} \left[ {s_{ab} } \right] + \sum\limits_{b \in B}^{{}} {\mathop {\hbox{max} }\limits_{a \in A} \left[ {s_{ba} } \right]} } }}{{n_{A} + n_{B} }}$$

where $s_{ab}$ and $s_{ba}$ are any pair-wise similarity measures between the *p*-dimensional element *a* of the set A and the *p*-dimensional element *b* of the set B. The term $\mathop {\hbox{max} }\limits_{b \in B} \;\left[ {s_{ab} } \right]$ is the maximum similarity between the element *a* of A and the set B and $\mathop {\hbox{max} }\limits_{a \in A} \;\left[ {s_{ba} } \right]$ is the maximum similarity between the element *b* of B and the set A. In other words, to calculate the Hausdorff-like similarity, for any element of a set, its maximum similarity to the other set is retained and the maximal contributions from all the elements of the set are summed up and then averaged on the total number of elements in both sets.

The name Hausdorff-like similarity is proposed to somehow recall the analogy with the calculation approach of the inner part of the original Hausdorff formula, which considers the minimum distance (or maximum similarity) between an element of a set and the other set. Unlike the Hausdorff formula, our measure finally accounts for the similarity contributions of all the set elements and the operators *sup* and *inf* are replaced by *max* and *min*, respectively, A and B being here only finite sets. Therefore, due to this difference, the proposed formula cannot be considered an outer measure and, instead of measuring the out boundary of the sets, it provides an overall average similarity.

In order to exemplify the Hausdorff-like similarity calculation, let A be a set including two elements A1, A2 (i.e., *n*_*A*_ = 2) and B a set with three elements B1, B2, B3 (i.e., *n*_*B*_ = 3). The first case we consider refers to the similarities *s* between all the possible pairs of elements of the two sets, which are collected in Table 1 and are supposed to be calculated from any *p* variables used to described the elements.

**Table 1:** Similarity matrix between the elements of sets A and B of the first case study; in the last column and row, the Hausdorff-like similarity contributions of all the individual elements are reported

	set B			$\bf \mathop {{max} }\limits_{b \in B} \;\left[ {s_{ab} } \right]$
	B1	B2	B3	
**set A**				
**A1**	1.00	0.20	0.35	1.00
**A2**	0.41	0.07	1.00	1.00
$\mathop {{\bf max}} \limits_{{\bf a} \varvec{\in} {\bf A}} \;\bf {\left[ {s_{ba} } \right]}$	1.00	0.20	1.00	

In this case, the sets A and B share two pairs of highly similar elements, that is, the pair A1–B1 (*s*_*A1,B1*_ = 1) and the pair A2–B3 (*s*_*A2,B3*_ = 1); however, the set B has an additional element (B2) that is quite dissimilar from both the elements of A (i.e., its maximal similarity is 0.20), then, the final *Hs* measure, which accounts for the similarity contributions of all the set elements, is:

$$Hs_{AB} = \frac{{\left( {1 + 1} \right) + \left( {1 + 0.20 + 1} \right)}}{2 + 3} = 0.84$$

Consider now the case of the pair-wise similarities between elements as reported in Table 2, where set A has only one element (A1). The Hausdorff-like similarity is:

$$Hs_{AB} = \frac{{1 + \left( {1 + 0.20 + 0.35} \right)}}{1 + 3} = 0.64$$

For the two examples above, if we applied the classical Hausdorff formula to measure the similarity between the sets, then we would obtain much lower similarity values, that is, 0.20 instead of 0.84 for the former example (Table 1) and 0.20 instead of 0.64 for the latter (Table 2). The approaches based on single and complete linkage (i.e., the maximum and minimum pair-wise similarity between the element pairs of the two sets, respectively) would give 1 and 0.07 for the first case (Table 1) and 1 and 0.20 for the second case (Table 2).

**Table 2:** Similarity matrix between the elements of sets A and B of the second case study; in the last column and row, the Hausdorff-like similarity contributions of all the individual elements are reported

	set B			$\bf \mathop {\hbox{max} }\limits_{b \in B} \;\left[ {s_{ab} } \right]$
	B1	B2	B3	
**set A**				
**A1**	1.00	0.20	0.35	1.00
$\mathop {{\bf max}} \limits_{{\bf a} \varvec{\in} {\bf A}} \;\bf {\left[ {s_{ba} } \right]}$	1.00	0.20	0.35	

Unlike the original Hausdorff formula and the complete linkage that mainly account for the analogy degree of the most different elements of the sets and the single linkage that instead accounts only for the most similar elements, our Hausdorff-like measure has been conceived to equally weigh both the presence of common/similar elements in the sets and that of different elements. When dealing with complex chemical systems like the sets of metabolites or chemical substructures generated from molecule dissection, chemical mixtures that are characterized by formulations of different substances, ionic liquids that are represented by their constituent ions, to quantify the analogy degree between two systems it is not appropriate accounting only for the common or different features but all the features of the systems have to be considered in the comparison. For instance, we cannot say that two molecules are identical (i.e., similarity of 1) if they generate one common metabolite but differ in the remaining molecular scaffold or two chemical mixtures are similar as they share a common substance but their formulations are basically different.

It is noteworthy that the proposed Hausdorff-like measure fulfils the *property of reflexivity* (or *identity*), that is, *Hs*_*AA*_ = 1. For the example reported in Table 3, the similarity measure is indeed calculated as follows:

$$Hs_{AA} = \frac{{\left( {1 + 1 + 1} \right) + \left( {1 + 1 + 1} \right)}}{3 + 3} = 1$$

**Table 3:** Similarity matrix between the elements of a set A; in the last column and row, the Hausdorff-like similarity contributions of all the individual elements are reported

	set A			$\bf \mathop {\hbox{max} }\limits_{a' \in A} \;\left[ {s_{aa'} } \right]$
	A1	A2	A3	
**set A**				
**A1**	1	Any	Any	1
**A2**	Any	1	Any	1
**A3**	Any	Any	1	1
$\mathop {{\bf max} }\limits_{{\bf a} \varvec{\in} {\bf A}} \;{\bf \left[ {s_{a'a} } \right]}$	1	1	1	

## Conclusions

All the basic properties required for a similarity measure hold for the Hausdorff-like measure:

$$\begin{array}{*{20}l} {1.} \hfill & {0 \le Hs \le 1} \hfill & {closure} \hfill \\ {2.} \hfill & {Hs_{AB} \ge 0} \hfill & {nonnegativity} \hfill \\ {3.} \hfill & {Hs_{AA} = 1} \hfill & {identity} \hfill \\ {4.} \hfill & {Hs_{AB} = Hs_{BA} } \hfill & {symmetry} \hfill \\ \end{array}$$

Moreover, it can be easily calculated, applied to any type of nonempty finite sets and could be a valuable alternative to the classical Hausdorff measure when all the parts of the systems to be compared are relevant to evaluate the analogy relationships. Advantages and drawbacks of the proposed measure in comparison with the classical measures for sets will be further investigated in a future paper.
